# Supplementary material for: Habituation effect in social networks as a potential factor silently crushing influence maximisation efforts
Source: Sci Rep. 2021 Sep 24;11:19055. doi: 10.1038/s41598-021-98493-9 (PMC8463708; doi:10.1038/s41598-021-98493-9)
Supplement: Supplementary file 1 — Supplementary Information. [file 41598_2021_98493_MOESM1_ESM.pdf]

Supplementary Information

Jaroslaw Jankowski

August 27, 2021

1 Coverage decrease for habituated processes for all used parameters

Table 1 shows percentage decrease of coverage of habituated processes based on Single Stage seeding when compared to non habituated setup for all used parameters including Propagation Probability (PP), Networks (NET), Seeding Percentage (SP), Seeding Strategy (ST) and different τ values.

2 Impact of habituation effect on spreading processes initialised by degree based and random seed selection

The impact of used seed selection strategies (degree-based and random) on coverage decrease was analyzed for each used Propagation Probability. It was shown that, for a very low PP with values 0.01 and 0.02, degree-based seed selection is more sensitive to the habituation effect, which is visible in Figure 1 (a) and (b). While the random selection intensity of the process is low, especially with low propagation probabilities, and the intensity of repeated messages is low, degree-based selection targets nodes with many connections, and the risk of repeated messages grows. Together with the increase in Propagation Probability, the performance of random seed selection increases, and with PP = 0.03, the number of configurations with a higher decrease is comparable for both strategies (Figure 1 (c)). Further increases in Propagation Probability result in a higher coverage decrease for random selection in most cases (Figure 1 (d) - (j). The situation changes starting from PP = 0.30, and a similar coverage decrease is observed with both strategies, as shown in Figure 1 (k) - (n).

3 Sequential Seeding performance for habituated setup represented by network coverage when compared to Single Stage Seeding

Table 2 shows increase of coverage of processes based on Sequential Seeding compared with Single Stage seeding for habituated setup for all used parameters including Propagation Probability (PP), Network (NET), Seeding Percentage (SP), Seeding Strategy (ST) and different τ values.

Illustrative example

A process was modelled, according to the proposed approach, as illustrated in Figure 2. The toy network was based on 9 nodes and 12 edges. All edges had assigned weights based on random values, in the range of (0,1), in both directions (e.g., from Node A to Node B and from Node B to Node A), with directions denoted by arrows. Assigned static weights were equivalent to random numbers generated during a stochastic process and typical Monte Carlo simulations. They were used for information flow modelling, which occurs if an edge value is lower than or equal to a given propagation probability PP. For example, with assumed propagation probability $PP = 0.3$, a message could spread from Node N1 to Node N9, due to the assigned weight of 0.29 in the N1 $\rightarrow$ N9 direction, but was not observed in the N9 $\rightarrow$ N1 direction, due to the assigned weight of 0.45 for that direction.

The states of nodes are denoted by colours: Active spreaders in red, newly activated nodes ready to activate neighbours in the next step in pink, failed activation and habituation effects in orange, active nodes with used

Table 1: **Coverage decrease for used parameters and different τ values**

Parameter	**Value**	**Difference**		**Decrease [%]**	**Decrease for each tau**							
		**H**	**p-value**		**1**	**2**	**3**	**4**	**5**	**10**	**15**	**20**
	0.01	1.19	<2.2e-16	17.57	17.57	17.7	17.84	17.43	17.57	17.57	17.57	17.57
	0.02	2.65	<2.2e-16	32.17	38.21	35.47	33.77	33.87	32.36	28.96	27.74	26.6
	0.03	5.06	<2.2e-16	41.93	50.44	49.09	45.91	44.97	42.61	37.07	33.69	31.67
	0.04	8.13	<2.2e-16	47.3	58.94	55.93	53.07	50.62	48.44	41.53	36.43	33.47
	0.05	10.64	<2.2e-16	48.64	63.3	58.56	56.14	53.47	50.24	40.79	35.32	31.39
	0.1	17.12	<2.2e-16	43.86	65.56	59.34	54.58	50.98	46.93	31.32	23.79	18.38
**PP**	0.15	16.99	<2.2e-16	35.01	57.28	52.28	47.06	41.41	36.85	21.19	13.62	10.38
	0.20	14.88	<2.2e-16	27.49	48.68	44.01	37.98	32.91	27.81	13.84	8.83	5.85
	0.25	12.08	<2.2e-16	20.59	36.16	34.25	29.57	24.61	20.74	9.51	5.75	4.12
	0.30	10.31	<2.2e-16	16.84	31.9	29.18	25.03	19.56	15.89	6.56	3.79	2.81
	0.35	8.80	<2.2e-16	13.88	28.95	25.48	19.99	15.27	11.85	4.69	2.87	1.98
	0.40	7.34	<2.2e-16	11.41	25.35	22.25	16.31	11.62	9.05	3.35	1.96	1.37
	0.45	5.93	<2.2e-16	9.16	22.21	17.87	12.54	8.75	6.63	2.57	1.5	1.19
	0.50	5.09	<2.2e-16	7.79	20.48	15.16	10.13	6.94	5.24	2	1.34	0.98
	N1	10.86	<2.2e-16	21.8	34.32	32.86	29.15	25.08	21.86	13.22	9.87	8.11
	N2	16.39	<2.2e-16	26.94	42.5	39.24	34.13	30.08	26.73	17.53	13.75	11.59
	N3	1.65	<2.2e-16	12.86	27.5	20.27	16.04	13.1	11.15	6.43	4.65	3.61
	N4	10.76	<2.2e-16	21.61	34.55	33.48	28.96	24.67	21.59	12.67	9.32	7.6
**NET**	N5	11.50	<2.2e-16	22.1	35.25	34.77	30.26	25.66	22	12.39	9.06	7.37
	N6	2.81	<2.2e-16	12.07	24.38	19.39	15.72	12.79	10.87	5.88	4.23	3.37
	N7	4.31	<2.2e-16	17.66	33.9	27.98	22.8	19	16.36	9.24	6.71	5.33
	N8	9.31	<2.2e-16	19.36	32.63	30.76	26.92	22.4	18.98	10.23	7.22	5.71
	N9	11.93	<2.2e-16	22.5	44.63	36.47	29.27	24.32	20.52	11.08	7.71	6.02
	N10	10.47	<2.2e-16	23.16	42.53	34.38	28.91	24.82	21.89	13.73	10.36	8.69
	0.01	8.83	<2.2e-16	23.63	38.26	36.3	31.78	27.14	23.58	13.79	10.04	8.2
	0.02	8.83	<2.2e-16	23.35	38.91	35.88	31.01	26.44	22.95	13.49	10	8.13
	0.03	8.62	<2.2e-16	22.84	38.49	35.38	30.07	25.77	22.27	13.13	9.67	7.94
**SP**	0.04	8.52	<2.2e-16	22.18	37.78	34.18	29.21	24.97	21.59	12.7	9.37	7.63
	0.05	8.47	<2.2e-16	21.79	37.7	33.46	28.64	24.32	21.06	12.36	9.23	7.52
	0.1	7.88	<2.2e-16	19.32	35.04	29.95	24.99	20.95	18.2	10.82	8.09	6.59
	0.15	7.29	<2.2e-16	16.99	31.76	26.28	21.72	18.29	15.83	9.39	7.01	5.73
**ST**	Random	8.88	<2.2e-16	21.95	36.23	33.49	29.12	24.9	21.73	12.88	9.53	7.76
	Degree	7.71	<2.2e-16	20.64	37.18	32.17	26.83	22.65	19.48	11.42	8.42	6.9

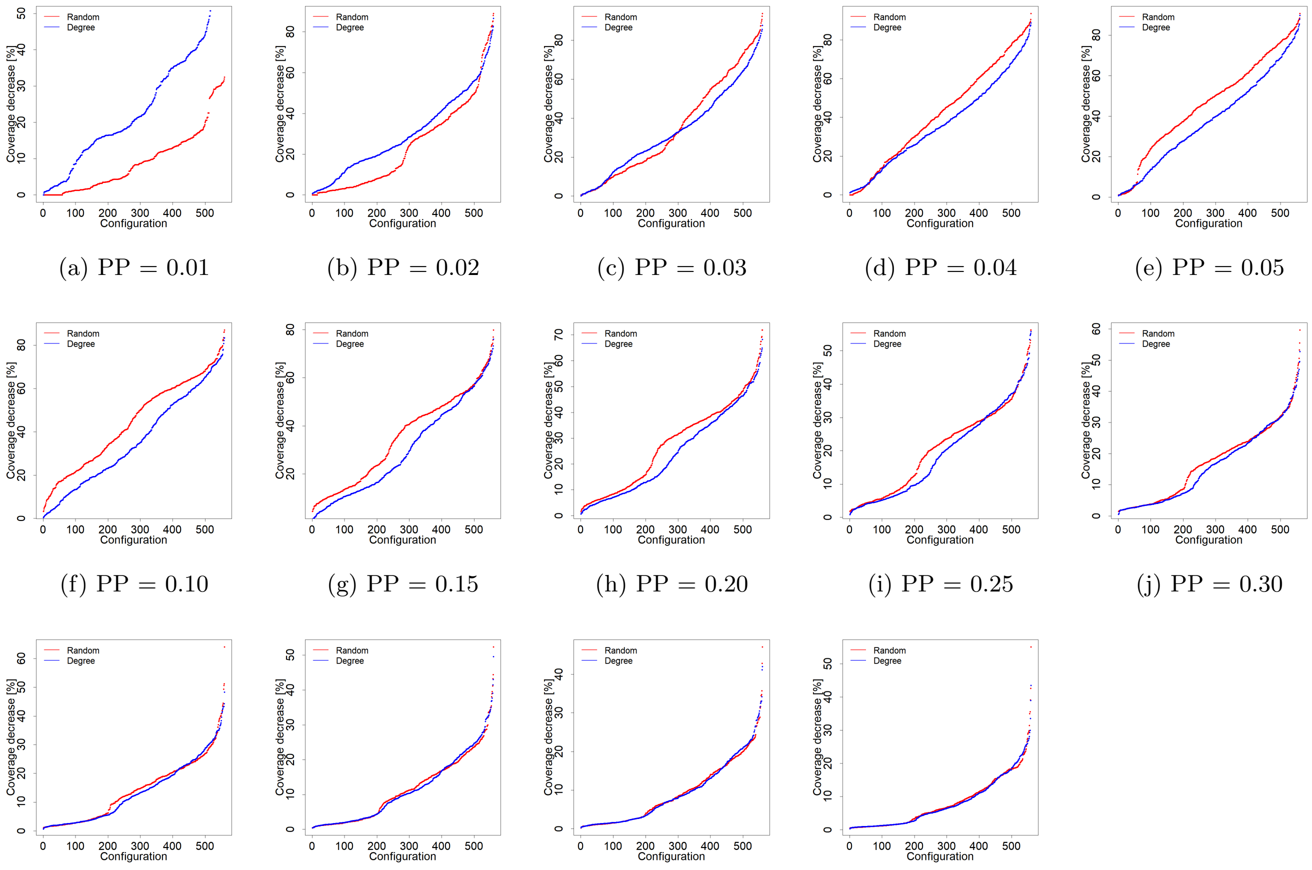

(a) PP = 0.01 (b) PP = 0.02 (c) PP = 0.03 (d) PP = 0.04 (e) PP = 0.05

(f) PP = 0.10 (g) PP = 0.15 (h) PP = 0.20 (i) PP = 0.25 (j) PP = 0.30

(k) PP = 0.35 (l) PP = 0.40 (m) PP = 0.45 (n) PP = 0.50

Figure 1: Difference of coverage decrease for degree based seed selection and random selection for all used Propagation Probabilities.

Table 2: **Performance of Sequential Seeding when compared to Single Stage Seeding with habituation taken into account for used τ values represented by coverage of SQ divided by coverage SS**

Parameter	**Value**	**Total coverage increase**			**Coverage increase for each τ**							
		**Increase**	**H**	**p-value**	**1**	**2**	**3**	**4**	**5**	**10**	**15**	**20**
	0.01	1.01	0.07	<2.2e-16	1.02	1.01	1.01	1.01	1.01	1.01	1.01	1.01
	0.02	1.14	0.73	<2.2e-16	1.23	1.18	1.16	1.16	1.14	1.1	1.09	1.08
	0.03	1.3	1.99	<2.2e-16	1.48	1.42	1.36	1.35	1.31	1.23	1.19	1.17
	0.04	1.42	3.46	<2.2e-16	1.72	1.62	1.54	1.5	1.45	1.32	1.24	1.21
	0.05	1.5	5.05	<2.2e-16	1.94	1.77	1.69	1.62	1.53	1.34	1.26	1.22
	0.1	1.52	10.86	<2.2e-16	2.31	2	1.82	1.7	1.59	1.28	1.19	1.15
**PP**	0.15	1.4	12.05	<2.2e-16	2	1.82	1.66	1.51	1.42	1.18	1.13	1.12
	0.20	1.3	11.31	<2.2e-16	1.74	1.6	1.46	1.36	1.28	1.13	1.11	1.1
	0.25	1.21	9.44	<2.2e-16	1.42	1.39	1.31	1.24	1.19	1.1	1.09	1.09
	0.30	1.17	8.68	<2.2e-16	1.35	1.31	1.25	1.18	1.14	1.09	1.09	1.08
	0.35	1.15	8.16	<2.2e-16	1.31	1.26	1.19	1.14	1.11	1.09	1.08	1.08
	0.40	1.13	7.63	<2.2e-16	1.26	1.23	1.16	1.11	1.1	1.08	1.08	1.08
	0.45	1.11	6.76	<2.2e-16	1.21	1.17	1.12	1.1	1.09	1.08	1.08	1.08
	0.50	1.1	6.44	<2.2e-16	1.19	1.14	1.1	1.09	1.08	1.08	1.07	1.07
	N1	1.22	8.18	<2.2e-16	1.41	1.37	1.31	1.25	1.21	1.12	1.1	1.09
	N2	1.25	10.16	<2.2e-16	1.51	1.45	1.35	1.28	1.23	1.13	1.1	1.09
	N3	1.29	3.04	<2.2e-16	1.28	1.28	1.27	1.28	1.28	1.29	1.3	1.3
	N4	1.22	8.23	<2.2e-16	1.4	1.38	1.3	1.24	1.21	1.13	1.11	1.1
**NET**	N5	1.21	8.34	<2.2e-16	1.42	1.4	1.32	1.25	1.2	1.1	1.09	1.08
	N6	1.16	3.75	<2.2e-16	1.3	1.23	1.19	1.16	1.14	1.11	1.1	1.1
	N7	1.21	4.21	<2.2e-16	1.45	1.33	1.26	1.21	1.19	1.13	1.12	1.12
	N8	1.19	6.81	<2.2e-16	1.35	1.32	1.27	1.21	1.17	1.11	1.1	1.09
	N9	1.17	6.61	<2.2e-16	1.48	1.33	1.23	1.18	1.14	1.08	1.07	1.07
	N10	1.15	4.71	<2.2e-16	1.37	1.23	1.17	1.14	1.12	1.09	1.08	1.08
	0.01	1.11	2.37	<2.2e-16	1.23	1.22	1.17	1.12	1.09	1.04	1.03	1.03
	0.02	1.14	3.62	<2.2e-16	1.32	1.28	1.21	1.16	1.13	1.06	1.05	1.05
	0.03	1.17	4.54	<2.2e-16	1.37	1.32	1.24	1.19	1.15	1.08	1.07	1.06
**SP**	0.04	1.19	5.34	<2.2e-16	1.4	1.34	1.26	1.21	1.17	1.1	1.08	1.08
	0.05	1.21	6.2	<2.2e-16	1.43	1.36	1.28	1.23	1.19	1.12	1.1	1.09
	0.1	1.27	9.64	<2.2e-16	1.51	1.41	1.33	1.28	1.24	1.18	1.16	1.15
	0.15	1.3	12.16	<2.2e-16	1.53	1.43	1.35	1.31	1.28	1.22	1.2	1.19
**ST**	Random	1.17	4.92	<2.2e-16	1.33	1.3	1.24	1.19	1.16	1.09	1.07	1.07
	Degree	1.24	7.5	<2.2e-16	1.48	1.39	1.3	1.25	1.21	1.15	1.13	1.13

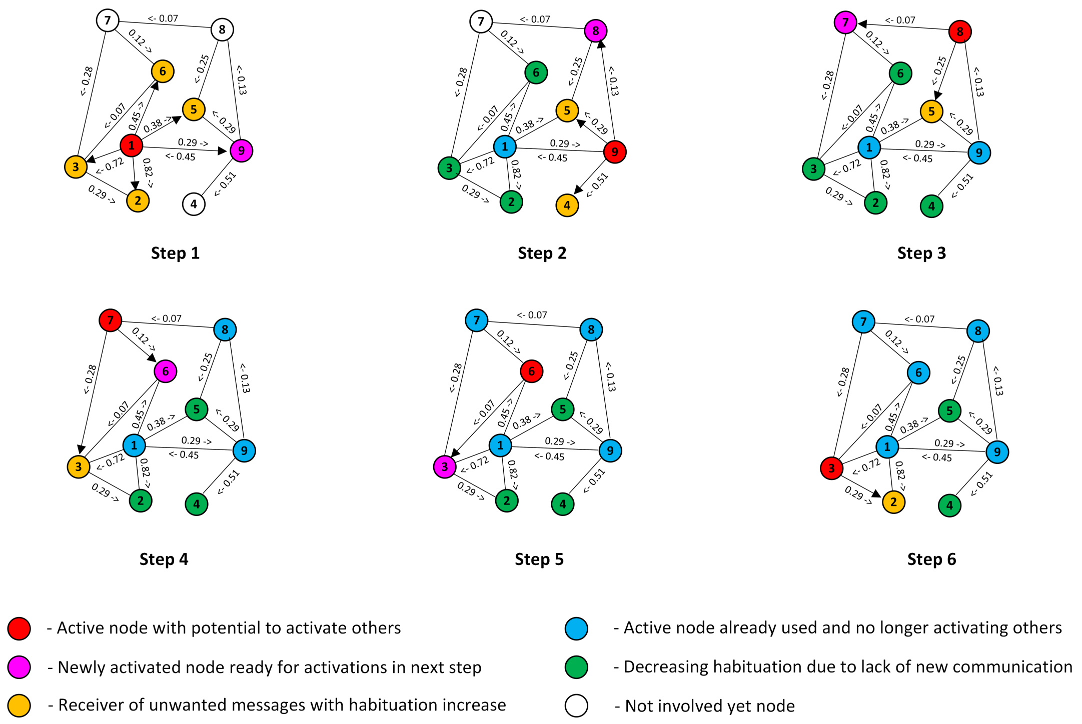

Figure 2: **(A)** Exemplary information-spreading process considering habituation effect, based on a toy network with 9 nodes and 12 edges.

spreading potential in earlier step and no more spreading in blue, and recovery mode from habituation (due to lack of contacts and ability to 'rest' from communication) in green.

At the beginning of the process, all nodes had assigned habituation with a value of 1.0, which means that the assumed propagation probability, PP, was used without reduction. If the responsiveness was changed to lower values, the propagation probability decreased; for example, if the habituation value for node i dropped in step j to $H_{i,j} = 0.5$, the propagation probability for node i would be $0.5 \times PP$.

Spreading processes, based on the ICM, were initiated by selecting from initial network nodes in the form of seeds, according to ranking, heuristics, or more complicated seed-selection methods used for influence maximisation. In the presented example, in Step 1 of the simulation, we selected Node N1 (red) as a seed node, due to its highest degree (based on it having five direct neighbours). It contacted Nodes N2, N3, N6, N5, and N9, and attempted to activate them. Due to the assumed propagation probability of $PP = 0.3$ and assigned weights in the proper direction (0.29), Node N9 was newly activated (pink), while attempts to activate the others failed. Failed attempts to activate represent a situation where target users receive currently unwanted information or content. In the future, they can be even more resistant to repeated stimuli, due to the habituation effect. With assumed parameters $\tau = 5$ and $\alpha = 1.05$, and according to Formula (1), the habituation factor of Nodes N2, N3, N5, and N6 was reduced to 0.82. The responsiveness for each node in each step is presented in Table 3. If any other attempts to send information from other nodes happen, the propagation probability drops from $PP = 0.3$ to $PP = 0.82 \times 0.3 = 0.25$. Step 1 of the simulation ended with new habituation factor values for the contacted nodes, denoted by orange. The habituation of Node N9 was unchanged, as it was no longer the target of any communication.

In Step 2, Node N1 did not take part in spreading (blue), because of the ICM; each activated node has only one chance to activate others. Node N9, activated in an earlier step, attempted to activate its inactive neighbours N4, N5, and N8. Communication with N4 failed, due to its weight being larger than PP and, so, its habituation factor decreased to 0.82. Communication with N5 initially had potential to be successful, as the weight assigned to the edge with the N9 $\rightarrow$ N5 direction was at the level of 0.29 but, due to the habituation factor assigned to Node N5 in Step 2 ($H_{5,2} = 0.82$), the propagation probability was reduced to 0.25 and transmission was not possible. Apart from this, the habituation of Node N5 increased, due to the next failed communication, where $H_{5,2} = 0.67$. Only one successful activation was performed, for Node N8. At the same time, Nodes N2, N3, and N6 received no

Table 3: Responsiveness level of Nodes N1–N9 for each simulation step, decreasing as habituation increased.

Node	Step 1	Step 2	Step 3	Step 4	Step 5	Step 6
1	1.00	1.00	1.00	1.00	1.00	1.00
2	0.82	0.85	0.88	0.90	0.92	0.74
3	0.82	0.85	0.88	0.70	0.70	0.70
4	1.00	0.82	0.85	0.88	0.90	0.92
5	0.82	0.67	0.55	0.64	0.71	0.76
6	0.82	0.85	0.88	0.88	0.88	0.88
7	1.00	1.00	1.00	1.00	1.00	1.00
8	1.00	1.00	1.00	1.00	1.00	1.00
9	1.00	1.00	1.00	1.00	1.00	1.00

communication from others and, so, they had the occasion to 'rest' from unwanted communication; thereby, their habituation factor recovered to 0.85, according to Formula (2).

In Step 3, Nodes N2, N3, and N6 in the second consequent period could 'rest' and, so, their responsiveness grew to 0.88. At the same time, the habituation factor of Node N4 recovered to 0.85, due to a lack of communication. Node N8 attempted to activate Node N5 and, similarly to the earlier step, activation failed due to the habituation of Node N5; its habituation factor decreased the propagation probability to $0.67 \times 0.3 = 0.20$. Additionally, N5 was again habituated, and its habituation factor dropped to 0.55. Node N8 successfully activated Node N7.

In Step 4, N7 activated N6, due to its weight being at the level of 0.12. N6 had the occasion to 'rest' during Stages 2 and 3 after the failed contact in Stage 1. Even with the habituation factor equal to 0.88, reducing propagation probability to a value of 0.26, it was still possible to transfer information. N7 also attempted to activate N3, but it had a habituation factor at the level of 0.88, and the reduced propagation probability made it impossible, even though the weight was equal to 0.29. Therefore, N3 was even more habituated, to the level of 0.7. Nodes N2, N4, and N5 were in 'rest' mode, and their habituation factor grew. Within Step 5, Node N6 activated N3, which was possible due to the low weight (of 0.07) still allowing for propagation, even when the probability was reduced to 0.21 after a reduction of 0.7×0.3.

In the last step, Node N3 failed to activate N2, due to the habituation of N2 with a factor of 0.92. Four steps of 'rest' did not allow for enough recovery to perform activation, even if the weight was equal to 0.29, allowing for transmission for full recovery. Node N5 was never activated, due to the habituation effect. Its initial weights with Nodes N8 and N9 were low enough for the assumed propagation probability PP, but the habituation factor reduced the PP, making activation impossible. The activation of Node N3 was delayed; instead of in Step 4, it was activated in Step 5.

4 Results for synthetic networks based on Barabási–Albert model

The analysis presented in this section includes results from simulations performed within networks generated with the use of the Barabási–Albert model and the degree distributions with the probability of observing degree x according to $p(x) = x^{-}\alpha$, with α representing a power law exponent. All networks had 1000 nodes and were generated with exponents starting from 1.5 to 2.50, with 0.1 increments, resulting in 11 networks. Simulations showed how a scale-free network topology affects differences between habituated and non-habituated spreading processes. Results shown in Figure 3 (a) illustrate a drop in differences between both processes for an increasing power law exponent α. Information spreading processes within networks with lower degrees and lower exponents for a scale-free model usually have lower dynamics and coverage. Additional slow-down as a result of habituation has a greater effect on the results. Figure 3 (b) shows a percentage decrease for each exponent and each consequent value until the level of 2.1. The highest difference was observed for α=1.5, with an average coverage decrease of 21.2%. Differences stabilize at the α level 2.2 with an 8% difference and a similar level until α=2.4. In terms of Propagation Probability and the impact of the habituation effect, results are presented in Figure 3 (c). The lowest differences are observed for low probabilities with values 0.01–0.05. Differences grow with a maximal difference of 6.04% observed for Propagation Probability 0.20. Differences are then lower until the level of 3.63%, observed for the highest used Propagation

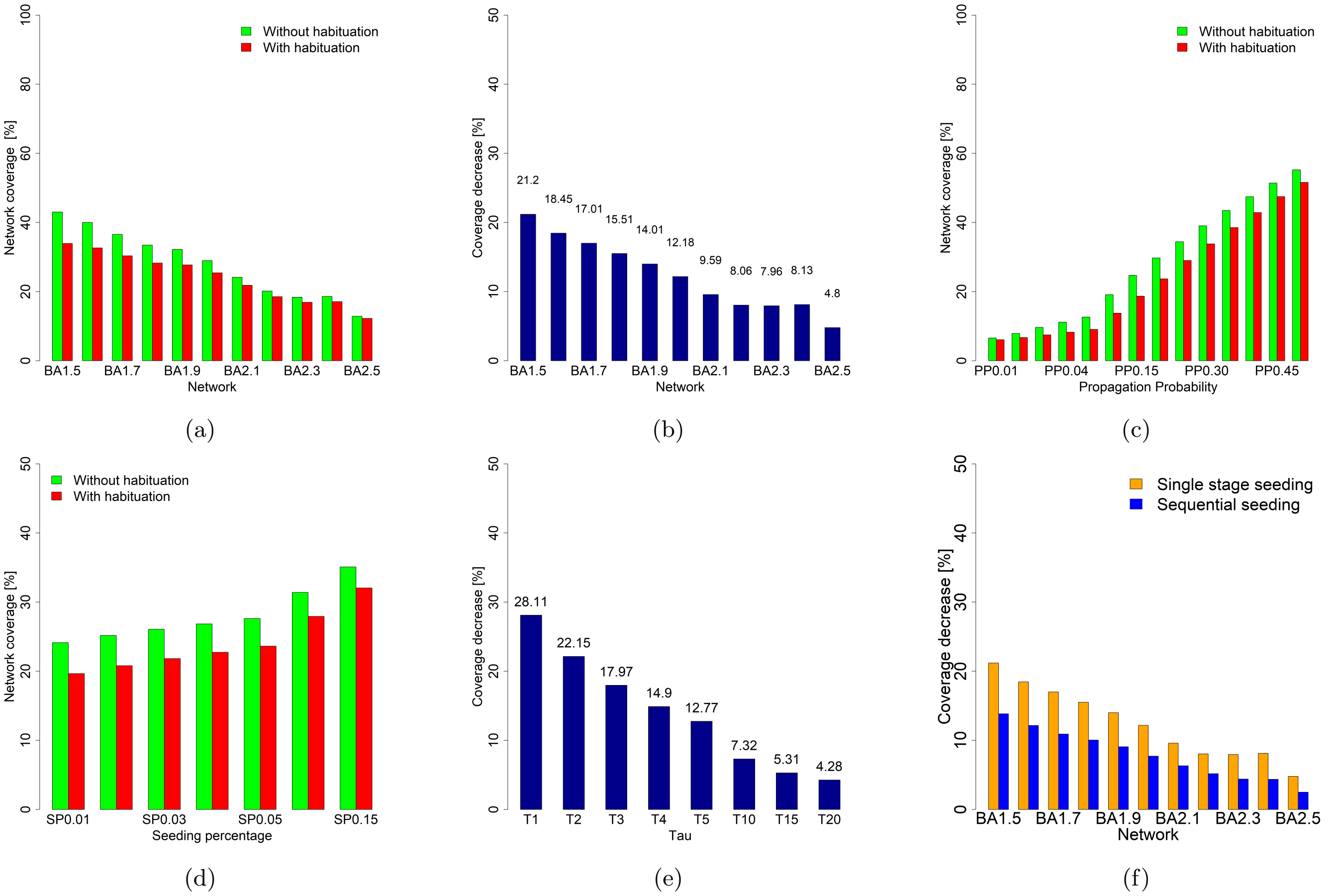

Figure 3: Coverage decrease for processes with taken into account habituation effect after comparison with non-habituated processes for BA networks with with exponent starting from 1.5 till 2.5 with 0.1 increment and (a) General differences, (b) Percentage decrease presented for all networks (c) Differences for used Propagation Probabilities (d) Differences for used Seeding Percentages (e) Differences for used τ values (f) Comparison for differences for Single Stage Seeding and Sequential Seeding within BA networks.

Probability equal to 0.50. Analysis for Seeding Percentage show that differences are highest at the level of 4.47% for the lowest Seeding Percentage with the value of 0.01. It drops until 3.02% for the highest values of Seeding Percentage (15%). Analysis of used τ values showed that the increase of τ by four times from T1 to T4 allowed for a reduction in the difference from 28.11% to 14.9%. Higher τ values increasing from T5 to T10 allowed for a change from 12.77% to 7.32%. Increasing τ twice from T2 to T4 allowed for a decrease from 22.15% to 14.9%. After comparison of Sequential Seeding with Single Stage Seeding, it was clear that Sequential Seeding is more resistant to the habituation effect. Average coverage for Single Stage seeding was at the level of 24.09% with habituation and 28.05% without. Average coverage for Sequential Stage seeding was at the level of 28.20% with habituation and 30.99% without.

5 Results for synthetic networks based on Erdos-Renyi model

Differences between habituated and non-habituated processes as well as the performance of sequential seeding were verified within random graphs according to the Erdos-Renyi model. Five networks were used with one thousand nodes each and from 1000 to 5000 edges generated with 1000 increments denoted by ER1, ER2, ..., ER5. In this approach, a given number of edges is selected in a uniform way from the collection of all possible edges. Results shown in Figure 4 (a) illustrate increased differences between both processes for an increased number of edges and, as a result, the average degree. Information spreading processes within networks with a low number of edges have low dynamics and

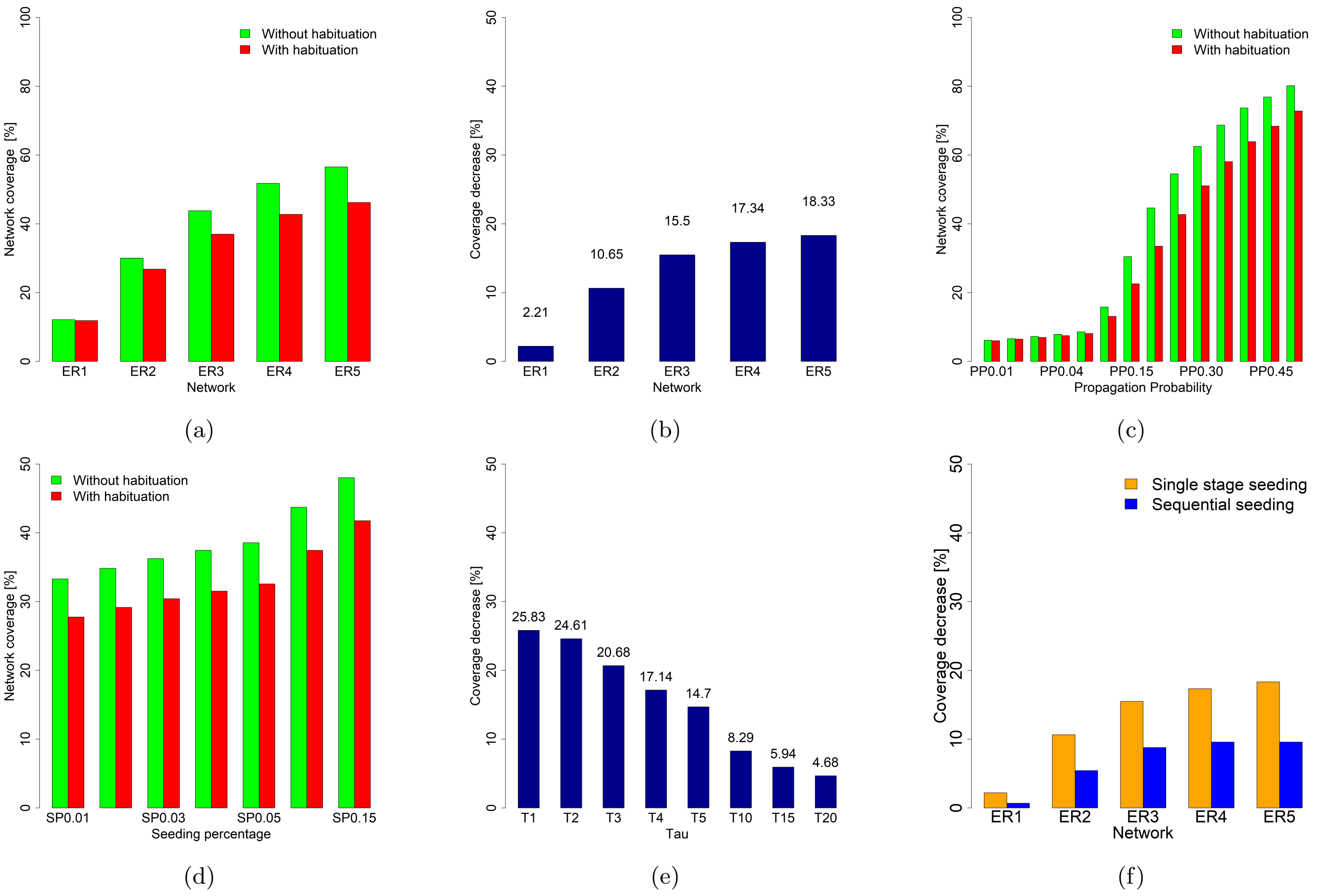

Figure 4: Coverage decrease for processes with taken into account habituation after comparison with non-habituated processes for ER networks with with number of edges within 1000 nodes based network starting from 1000 till 5000 with 1000 increment and (a) General differences between habituated and non-habituated process are presented, (b) Percentage decrease presented for used networks (c) differences for all used Propagation Probabilities (d) Differences for all seeding percentages (e) Differences for all used τ values (f) Comparison for differences for Single Stage Seeding and Sequential Seeding.

coverage even without a habituation effect. An additional habituation effect slightly changed the results. Results in Figure 4 (b) show a 2.21% drop in coverage. Differences increased together with increased network density, and a substantial increase was observed for the networks ER2 and ER3 with 10.56% and 15.5%. A further increase in the number of edges resulted in higher differences at the level of 17.34 and 18.33 for the networks ER4 and ER5, but the increase when compared to ER3 was not substantial. For the used Propagation Probabilities from 0.01 to 0.50, the effect of habituation is presented in Figure 4 (c). Low differences are visible for low probabilities. Differences increase from PP=0.05. A maximal difference of 11.84% was observed at PP=0.25. Starting from PP=0.30, differences were lower, and when PP has at the highest used value of 0.50, the difference was at the level of 7.39%. Analysis of results for Seeding Percentage show a growth of differences together with a growing number of seeds. The Highest Seeding Percentage obtained was a 6.26% difference, while the lowest was 5.51%. For the analyzed τ values, τ=1 resulted in a 25.83% decrease. The lowest decrease at the level of 4.68% was observed for τ=20. Analysis of the performance of Sequential Seeding when compared to Single Stage under the habituated setup showed substantial differences. The coverage decrease in random networks was at the level of 6.81% for Sequential Seeding and 12.80% for Singe Stage seeding. The highest differences were observed for the network ER1 with a 2.20% decrease for Single Stage Seeding and 0.68% for Sequential Seeding. Networks with a higher density such as ER4 and E5 still resulted in substantial differences and a coverage decrease for Sequential Seeding of 9.59 and 9.58; for Single Stage Seeding, this was at the level of 17.34% and 18.33%.

6 Results for synthetic networks based on Watts-Strogatz model

Other simulations were performed within WS networks. The same size of 1000 nodes, as for BA and ER networks, was used. During synthetic network generation, two parameters were used. First, a number of connections was denoted from each vertex. Values of 1–5 were used. The second parameter represented the rewiring probability within an initial regular lattice with values 0, 0.025, 0.50, 0.75, and 1. A combination of all parameters resulted in 25 networks denoted as WS_{ij}, with i representing the number of connected neighbours and j representing the rewiring probability. Results in Figure 5 (a) showed that the main differences are related to a number of connected neighbours rather than rewiring probabilities. Figure 5 (b) shows that the results for only one connected neighbour show a very low coverage, even without a habituation effect. Habituation further decreases coverage, but a minimal decrease of 0.19% was observed for the rewiring probability equal to 0.00. The maximal decrease of 2.4% is visible for the rewiring probability equal to 1.0. The increase in the number of connected neighbours resulted in a substantial coverage increase. The habituation effect decreases coverage even by 11.33% for a maximal rewiring probability. However, even for a lower rewiring probability such as 0.50 or 0.75, a decrease was observed at the level of 10.25% and 10.7%. For a higher number of neighbours, i.e., 3, 4, and 5, the highest decrease of coverage with a habituation effect was observed for a zero rewiring probability. The results for other rewiring probabilities are similar for each group of results with the same number of neighbours with a level of 15%, 16%, and 18%. Analysis for Propagation Probabilities within a range from 0.01 to 0.50, shown in Figure 5 (c), shows low differences for low probabilities. Differences grow starting from PP=0.10. The maximal difference of 11.15% was observed for PP=0.35. Starting from PP=40, differences are lower, and when PP has the highest value of 0.50, the difference achieves a level of 8.50%. This pattern is similar to that of BA and ER networks. For the Seeding Percentage, with results shown in Figure 4 (d), differences are smaller for a low seeding percentage with a value of 5.42% from SP=0.01 to 6.01% for the highest Seeding Percentage of 0.15. For the value of τ=1, Figure 5 (d) shows a 28.68% decrease. The lowest decrease at the level of 4.91% was observed for τ=20. Analysis of the performance of Sequential Seeding when compared to Single Stage Seeding under a habituated setup showed substantial differences, shown in Figure 5 (f). The coverage decrease within networks with one neighbour contacted was at low levels for Single Stage Seeding and for Sequential Seeding. For a higher number of neighbours, Sequential Seeding delivered worse results for a rewiring probability equal to 0.00 and a number of neighbours equal to 2, 3, or 4. For other rewiring probabilities, Sequential Seeding delivered a lower decrease than did Single Stage Seeding.

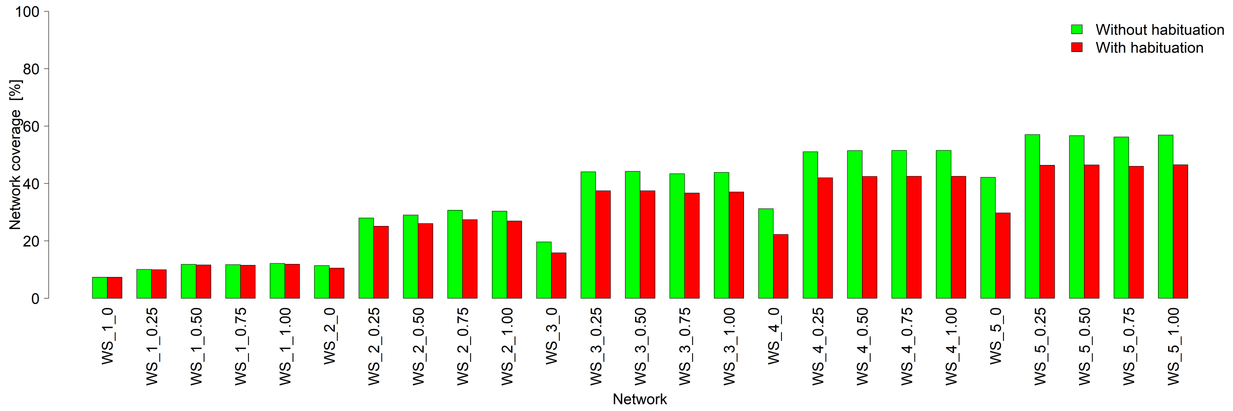

(a)

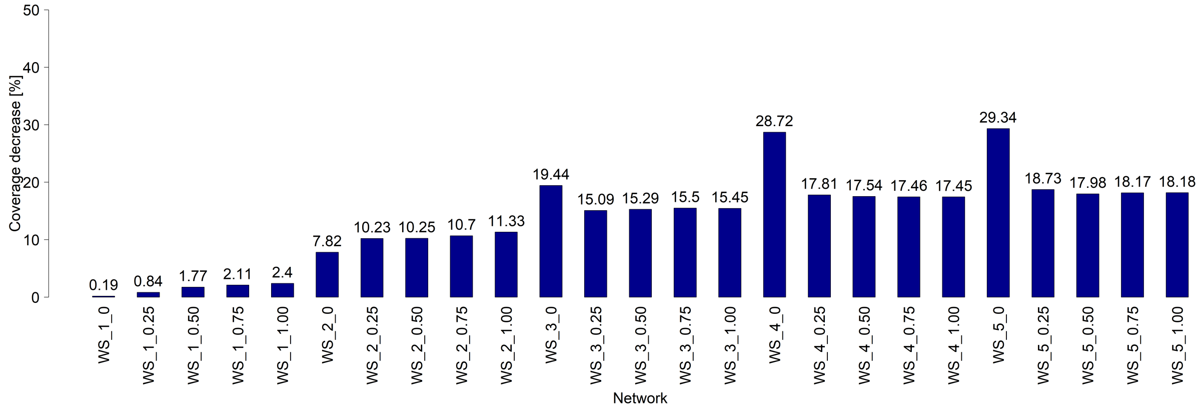

(b)

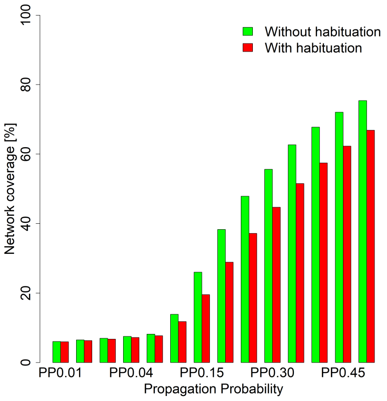

(c)

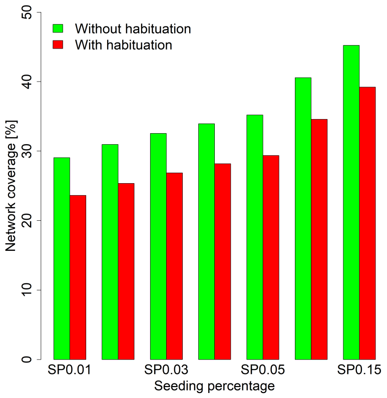

(d)

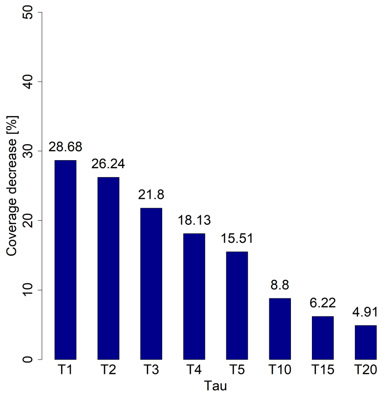

(e)

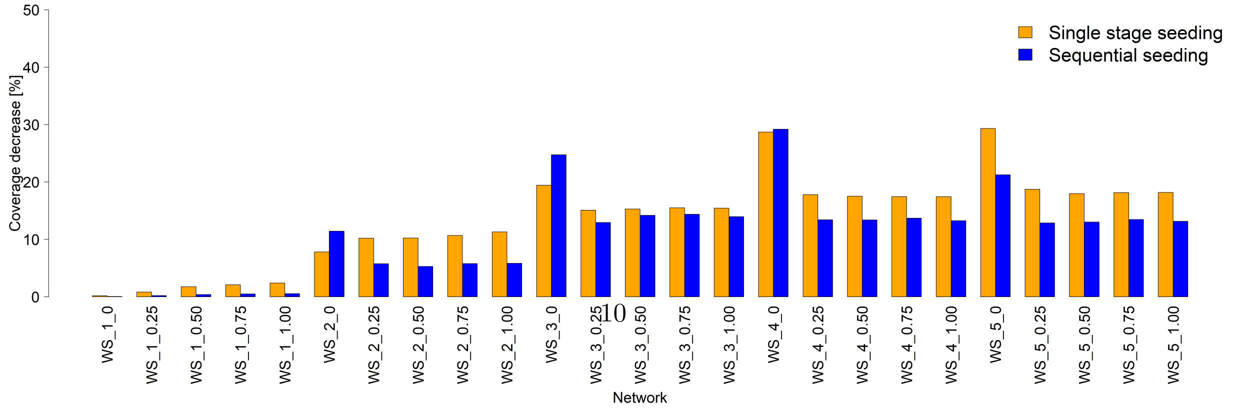

(f)

Figure 5: Coverage decrease for processes with taken into account habituation after comparison with non-habituated processes for WS networks based on 1000 and connected neighbours with range 1-5 and rewiring probability from 0.00 till 1.00 with 0.25 interval presented in figures: (a) General differences between habituated and non-habituated
